# Trends and factors associated with skilled birth attendance in a post-Ebola context: DHS Guinea 2018

**DOI:** 10.4102/jphia.v16i1.512

**Published:** 2025-01-16

**Authors:** Madeleine Toure, Fanta Barry, Tiany Sidibe, Sadan Camara, Ramata Diallo, Kaba Saran Keita, Maimouna Balde, Bienvenu Salim Camara, Karifa Kourouma, Mamadou Dioulde Balde

**Affiliations:** 1 Center for Research in Reproductive Health in Guinea (CERREGUI), Conakry, Guinea; 2 Department of Medidine, Faculty of Health Sciences and Techniques, Gamal Abdel Nasser University of Conakry, Conakry, Guinea

**Keywords:** skilled birth attendance, epidemics, post-Ebola, women aged 15–49, Guinea

## Abstract

**Background:**

In Guinea, the 2013–2015 Ebola epidemic profoundly affected maternal health service use. The frequency of births attended by skilled health professionals in the post-Ebola context remains under-documented.

**Aim:**

The aim of this study was to analyze the trend and factors associated with skilled births among women aged 15-49 between 2016 and 2018 in Guinea.

**Setting:**

The Republic of Guinea was the setting for this study.

**Methods:**

Data from 3018 women aged 15–49 years who had at least one live birth over the period 2016–2018 were analysed. The simple binary logistic regression model was used to analyse factors associated with skilled births using Stata software version 16.1. The significance level was set at 5%.

**Results:**

Our study found that 57.3% of deliveries were skilled births. This proportion showed a remarkable variation with a trend in assisted deliveries from 61% in 2016 to 59% in 2017 and then to 50% (*p* = 0.003) in 2018. Factors associated with skilled birth attendance in post-Ebola were: having no level of education (odds ratio [OR] = 0.39; confidence interval [CI]: 0.31–0.77), performing four or more antenatal care (ANC) (OR = 12.10; CI: 8.24–17.77), residing in a rural area (OR = 0.25 [0.17–0.37]), having a spouse who was a trader or manual worker, belonging to a household with an intermediate or poor wealth index and residing in the Labé region.

**Conclusion:**

This study showed that the proportion of assisted births showed a downward trend between 2016–2018.

**Contribution:**

The interventions undertaken to strengthen the maternal health system in the aftermath of the Ebola epidemic should be reinforced and maintained, in particular the retention of health providers deployed in rural areas and capacity building (training, equipment) for community health workers would help to improve this indicator

## Introduction

Every hour in 2020, around 33 women worldwide died for reasons related to pregnancy or childbirth.^1^ The main causes of these deaths were obstructed labour, sepsis, haemorrhage and eclampsia.^2^ A significant proportion – 95% – of these deaths occur in low- and middle-income countries,^1^ because of a lack of skilled birth attendance during childbirth.^3,4^ According to estimates made by the World Health Organization (WHO) in 2017, maternal mortality was 576 per 100 000 live births in Guinea, despite the implementation of programmes aimed at reducing maternal mortality.^5,6^

Skilled birth attendance leads to better pregnancy outcomes and contributes to improved maternal and newborn survival.^7^

Between 1990 and 2015, skilled birth attendance contributed to a 44% reduction in the global maternal mortality trend, from 385 deaths per 100 000 live births to 216 deaths per 100 000 live births.^8^ Despite this progress, around 34% of births worldwide are assisted by traditional birth attendants,^7^ indicating that 45 million births take place at home without skilled health professionals every year.^7^ In 2017, 50% of births were attended by skilled health professionals in Africa.^9^ Previous studies have reported varying frequencies of skilled birth attendance in different settings.

For example, studies conducted in New Guinea in 2021^3^ and in Bangladesh in 2022^10^ reported respective proportions of skilled birth attendants of 53.4% and 57.6%. However, this proportion remains low in some African countries such as Nigeria (23%) and Zambia (37%).^11^ According to Guinea’s Demographic and Health Survey (DHS), the proportion of skilled births was 55% in 2018. For some studies, education level, wealth index, distance from the health facility, residence and achieving an antenatal care (ANC) visit were factors associated with skilled birth attendance.^11,12,13,14^

In Guinea, the introduction of free obstetric care in 2010 helped to improve reductions of unmet needs for obstetric care, including ANC, assisted delivery and caesarean section.^15^ However, the occurrence of the Ebola epidemic between 2013 and 2015 led to a reduction of the use of maternal health services.^16^ A study conducted in Sierra Leone showed a reduction of more than 20% in the number of deliveries and caesarean sections during the Ebola epidemic.^17^ Furthermore, this reduction was linked to the closure of many health centres and the fear or lack of trust of the communities towards health professionals but also to go to maternal health facilities.^16,18^ Most of these studies focused on the immediate effects of the Ebola epidemic in the most affected areas.^15,18^ Very few studies have assessed the changing trend and factors associated with skilled birth attendance after the Ebola outbreak at the national level. To contribute to the resolution of this issue, we intend through this study to answer the following question: What are the annual trends and factors associated with skilled birth attendance between 2016 and 2018 in Guinea?. This study aims to analyse the evolution of proportions and identify factors associated with childbirth assisted by skilled health personnel among women aged 15–49 years between 2016 and 2018 in Guinea. The results of this study will make it possible to propose solutions aimed at improving the availability of assisted childbirth services post-epidemic and to guide policy makers in their efforts to ensure the post-epidemic resilience of the country’s maternal health system.

## Materials and methods

### Setting

Guinea is located in West Africa and covers an area of 245 857 km^2^. According to the projection of the RGPH3, the Guinean population was estimated at 13 million in 2018. The country is composed of seven administrative regions, with Conakry as the capital. The country’s socio-economic situation is marked by persistent poverty. According to data from the 2019 Global Vulnerability Analysis for Guinea, 55.2% of the population was living below the poverty line in 2019.^19^ In terms of reproductive health, Guinea is one of the countries where the total fertility rate (TFR) is still high, at 4.8 children per woman.^20^ According to the 2018 DHS, the proportion of skilled births was 55%. Maternal health services are provided at the primary, secondary and tertiary levels of the health system. At the community level, the provision of maternal and child health services is supported by community health workers and community relays who raise awareness and refer women to health facilities.

### Study design and period

This was a secondary analysis of data from the most recent Guinea DHS collected from 27 March 2018 to 28 June 2018. The DHS is a nationally representative cross-sectional survey. The methods for data collection as well as the reports are available and accessible on the DHS programme website (http://dhsprogram.com). This study used data from the individual women’s questionnaire.

### Study population and sample

**FIGURE 1:**
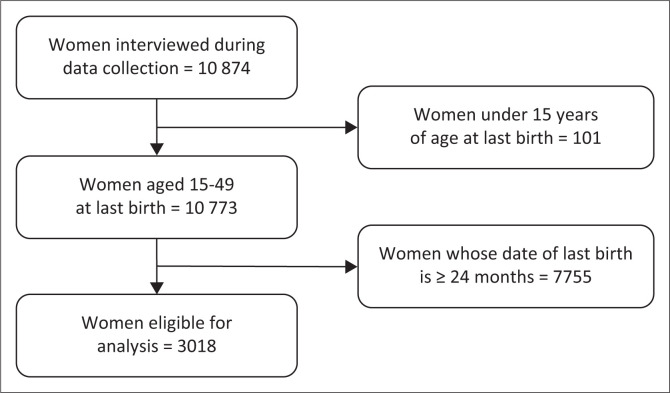
Women’s selection chart.

We considered as the study population all women aged 15–49 years from the individual women’s questionnaire. Two-stage weighted stratified random sampling, representative at the national level and at the level of the residential areas, was used in the framework of the EDS V 2018. We specifically analysed the data of women who had at least one live birth during the period 2016–2018. For women who had more than one live birth during this period, the most recent birth was retained. Out of a total of 10 874 women interviewed during the EDS V collection, 3018 women were eligible for our analysis after excluding women aged under 15 years and children whose birth age was greater than or equal to 2 years because we assumed that these births occurred during or before the Ebola epidemic (Figure 1).

### Study variables

#### Dependent variable

The dependent variable for this study was skilled birth attendance. Women who had a live birth were asked whether their delivery had been assisted by a doctor, nurse, midwife or assistant nurse. Those who answered ‘Yes’ to at least one of the questions concerning these three types of health personnel were considered to have had a skilled birth. The answer ‘Yes’ was coded as ‘1’ and ‘No’ as ‘0’.

#### Independent variables

The independent variables assessed were age, women’s level of education, men’s level of education, wealth index, women’s occupation, men’s occupation, residence, marital status, administrative region, household size, women’s autonomy in decision-making about their healthcare, media exposure, distance of residence from health facility and place of delivery. The choice of these variables was based on the literature^10,21,22^ (Table 1).

**TABLE 1:** List of study variables.

Variables	Modalities
**Women’s age**	1 = 15 to 24 years old
	2 = 25 to 34 years old
	3 = 35 to 49 years old
**Women’s education level**	1 = Secondary/ Higher
	2 = Primary
	0 = None
**Education level of the women’s partner**	1 = Secondary/ Higher
	2 = Primary
	0 = None
**Women’s profession**	1 = Civil servant
	2 = Trader/other
	0 = No profession
**Profession of the women’s partner**	1 = Civil servant
	2 = Trader/other
	0 = No profession
**Distance to the health facility perceived as a problem**	1 = Yes
	2 = No
**Wealth index**	1 = Rich
	2 = Intermediate
	3 = Poor
**Number of ANC performed**	1 = ≥ 4 ANC
	2 = 1–3 ANC
	0 = None
**Media exposure**	0 = No
	1 = Yes
**Place of residence**	1 = Urban
	2 = Rural
**Region of residence**	1 = Conakry
	2 = Boké
	3 = Faranah
	4 = Kankan
	5 = Kindia
	6 = Labé
	7 = Mamou
	8 = Nzérékoré

ANC, antenatal care.

### Data processing and analysis

Stata software version 16.1 was used to process and analyse the data. Descriptive data were analysed as proportions with 95% confidence intervals (CI) or means with standard deviations. The proportion of women with skilled attendance at birth was presented by birth place and by region.

The annual trend in the proportion of skilled birth attendants was described as an epidemic curve.

The Pearson Chi-square test was used to compare the variables, with a significance level of 5%.

To determine the factors associated with skilled birth attendance, a multivariate logistic regression model was used. Crude and adjusted odds ratios (OR) and 95% CI were presented. The variables to be included in the multivariate analysis were determined by a univariate analysis between the dependent variable and the potential explanatory variables. The inclusion threshold of 0.20 was used.

We used a bottom-up, step-by-step modelling procedure: starting with the empty model, we added the significant variables one by one (the most significant first) to the univariate analysis. When moving from one model to the next, the Wald Test was used to assess the significance of the variable introduced. If the variable was significant at the 5% threshold, it was retained in the model and the next variable was introduced. In the opposite case (variable introduced not significant in the model), the variable was withdrawn.

The final model was subjected to a global fit test (Hosmer-Lemeshow test) and a specification test (link test). The Stata ‘survey’ package was used for this purpose.

### Ethical considerations

Demographic and Health Surveys are subject to review by ethics committees before the survey is carried out. Anonymity and confidentiality were respected during primary data collection. Written informed consent was sought from participants before data collection began. An authorisation request was made and obtained online via the DHS platform (https://dhsprogram.com) from the DHS database management programme (DHS-Programme). The ethical standards applicable to research without direct contact with human or animal subjects are respected and applied in demographic and health surveys.

## Results

### Socio-demographic characteristics of sampled women

The average age of the women was 28.2 years, with a standard deviation of ± 0.08 (Table 2). Of these, 38.2% were aged between 25 and 34, and the majority (74.1%) had no formal education. In terms of occupation, more than half the women were shopkeepers or manual workers (61.4%) and almost all (94.2%) were married. Nearly half (49%) of the women had attended between one and three ANC visits, and most of them lived in rural areas (71.4%) (Table 2).

**TABLE 2:** Socio-demographic characteristics of women aged 15–49 who had had at least one birth (*N* = 3018).

Variables	*n*	Percentage
**Women’s age group (years)**		
15–24	1062	35.9
25–34	1162	38.2
35–49	794	25.9
Mean age ± standard deviation	28.2 ± 0.08	
**Women’s education level**		
Secondary/Higher	405	13.7
Primary	370	12.2
None	2243	74.1
**Education level of the women’s partner**		
Secondary/Higher	557	20.3
Primary	220	7.7
None	2035	72.0
**Women’s profession**		
Civil servant	340	11.3
Trader/other	1819	61.4
No profession	846	27.3
**Profession of the women’s partner**		
Civil servant	553	19.3
Trader/other	2110	75.4
No profession	149	5.3
**Women’s marital status**		
Married/in union	2848	94.2
Divorced/Widowed	51	1.7
Single	119	4.1
**Number of ANC performed**		
None	384	12.7
1–3 ANC	1486	48.9
≥ 4 ANC	1148	38.4
**Media exposure**		
Yes	1145	38.9
No	1873	61.1
**Household size (member)**		
Less than 7	1266	42.5
7 or more	1752	57.5
**Distance to the health facility perceived as a problem**		
No	1506	48.2
Yes	1512	51.8
Place of delivery		
Health facility	1587	54.1
Home	1431	45.9
**Women’s autonomy in healthcare decision-making**		
Yes	250	9.1
No	2598	90.5
**Socioeconomic status**		
Poor	1020	34.0
Intermediate	595	19.4
Rich	1403	46.6
**Place of residence**		
Urban	853	28.6
Rural	2165	71.4
**Regions of residence**		
Conakry	249	11.0
Mamou	289	7.3
Nzerekore	353	14.6
Labé	380	11.9
Kindia	404	15.1
Boké	417	10.3
Faranah	423	11.0
Kankan	503	18.9

ANC, antenatal care.

### Proportion and trends in the proportion of skilled births

**FIGURE 2:**
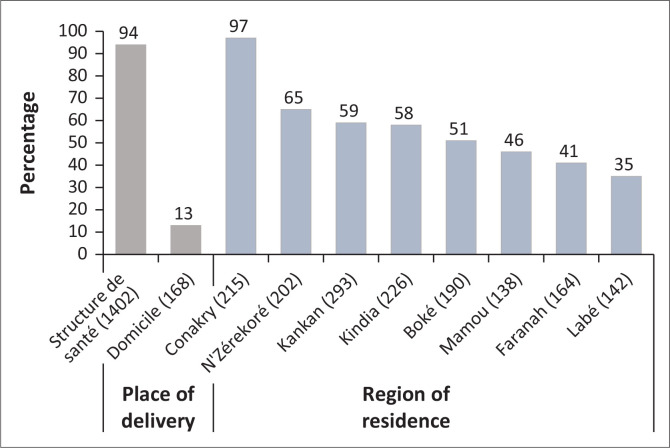
Proportion of births attended by place of delivery and region of residence.

**FIGURE 3:**
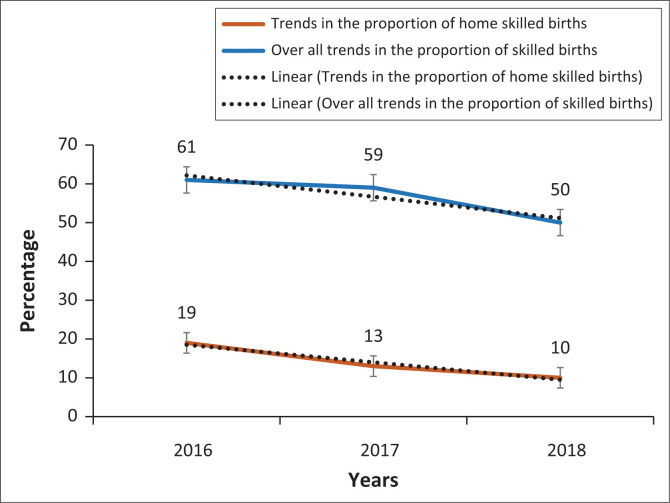
Annual trends in proportions of skilled births (2016–2018).

Countrywide, 57.3% (95% CI [53.7 – 60.8]) of women had received a birth attended by skilled health personnel between 2016 and 2018. Distributed by region and place of delivery, this proportion varied from 35% for the Labé region to 97% for the city of Conakry (*p* < 0.001). Also, the proportion of home deliveries with the assistance of qualified health professionals was 13% (95% CI [10.7 – 15.7]) (Figure 2). The trend in the proportion of births attended by skilled professionals decreased each year between 2016 and 2018, from 61%, 59% and 50%, respectively (*p* < 0.001) (*p* < 0.001). The proportion of skilled home births also had a decreasing annual trend from 19% in 2016 to 13% in 2017 and 10% in 2018 (*p* < 0.001) (Figure 3).

### Factors associated with skilled birth attendance

Table 3 shows women aged 15–49 years who had at least one live birth between 2016 and 2018 in Guinea. In univariate analysis, these are the factors associated with skilled birth attendance were women’s age, women’s and men’s level of education, women’s and men’s occupation, number of ANC performed, media exposure, distance from facility perceived as a problem, household wealth index, area of residence and region of residence.

**TABLE 3:** Factors associated with skilled birth attendance among women who had at least one birth between 2016 and 2018 in Guinea.

Variables	Crude OR	95% CI	*p*	Adjusted OR	95% CI	*p*
**Women’s age group (years)**						
35–49	Ref.	Ref.	-	Ref.	Ref.	-
25–34	1.16	0.95 – 1.41	0.146	0.92	0.74 – 1.17	0.525
15–24	1.30*	1.02 – 1.65	0.031	0.95	0.74 – 1.21	0.669
**Women’s education level**						
Higher/Secondary	Ref.	Ref.	-	Ref.	Ref.	-
Primary	0.29*	0.08 – 0.17	< 0.001	0.67	0.41 – 1.09	0.112
None	0.12*	0.18 – 0.46	< 0.001	0.39*	0.31 – 0.77	< 0.001
**Education level of women’s partner**						
Higher/Secondary	Ref.	Ref.	-	Ref.	Ref.	-
Primary	0.18*	0.14 – 0.25	0.003	-	-	-
None	0.33*	0.21 – 0.51	< 0.001	-	-	-
**Women’s profession**						
Civil servant	Ref.	Ref.	-	Ref.	Ref.	-
Trader/workwoman	0.35*	0.25 – 0.49	< 0.001	-	-	-
No profession	0.49*	0.33 – 0.72	< 0.001	-	-	-
**Profession of Women’s partner**						
Civil servant	Ref.	Ref.	-	-	-	-
Trader/workman	0.23*	0.18 – 0.29	< 0.001	0.56*	0.42 – 0.74	< 0.001
No profession	0.33*	0.20 – 0.54	< 0.001	0.71*	0.43 – 1.17	0.177
**Number of ANC performed**						
None	Ref.	Ref.	-	Ref.	Ref.	-
1–3 ANC	6.92*	4.66 – 10.27	< 0.001	6.60*	4.58 – 9.50	< 0.001
≥ 4 ANC	17.93*	11.74 – 27.37	< 0.001	12.10*	8.24 – 17.77	< 0.001
**Women’s autonomy in healthcare decision-making**						
Yes	Ref.	Ref.	-	-	-	-
No	0.77	0.56 – 1.06	0.120	-	-	-
**Media exposure**						
Yes	Ref.	Ref.	-	Ref.	Ref.	-
No	1.76	1.45 – 2.14	< 0.001	0.88	0.63 – 1.12	0.420
**Distance to the health facility perceived as a problem**						
No	289	2.30– 3.61	< 0.001	1.20*	0.98 – 1.47	0.074
Yes	Ref.	Ref.	-	-	-	-
**Household size (people)**						
Less than 7	Ref.	Ref.	-	-	-	-
7 or more	1.03	0.87 – 1.22	0. 717	-	-	-
**Household wealth index**						
Rich	Ref.	Ref.	-	Ref.	Ref.	-
Intermediate	0.13	0.09 – 0.19	< 0.001	0.43*	0.31 – 0.59	< 0.001
Poor	0.07	0.05 – 0.09	< 0.001	0.29*	0.22 – 0.41	< 0.001
**Place of residence**						
Urban	Ref.	Ref.	-	Ref.	Ref.	-
Rural	0.05	0.03 – 0.07	< 0.001	0.25*	0.17 – 0.37	< 0.001
**Region of residence**						
Boké	0.03	0.01 – 0.08	< 0.001	0.42*	0.19 – 0.92	0.030
Conakry	Ref.	Ref.	< 0.001	Ref.	Ref.	-
Faranah	0.02	0.01 – 0.05	< 0.001	0.32*	0.15 – 0.69	0.004
Kankan	0.05	0.02 – 0.12	< 0.001	0.63*	0.29 – 1.32	0.222
Kindia	0.04	0.01 – 0.10	< 0.001	0.46	0.18 – 1.20	0.113
Labé	0.02	0.01 – 0.04	< 0.001	0.19*	0.07 – 0.54	< 0.001
Mamou	0.03	0.01 – 0.06	< 0.001	0.48*	0.22 – 1.06	0.072
N’zerekore	0.06	0.02 – 0.14	< 0.001	1.12*	0.52 – 2.40	0.766

OR, odds ratio; CI, confidence interval; ANC, antenatal care; Ref., reference group.

* the factor was statically associated with the dependent variable with a *p* < 0.05.

In multivariate analysis, only women’s level of education, men’s occupation, household wealth index, place of residence and region were significantly associated with birth attended by skilled health personnel.

Women with no formal education were 61% less likely to give birth with a skilled birth attendant than women with secondary education or higher (adjusted OR = 0.39; CI = [0.31–0.77]).

Similarly, women living in rural areas were 75% less likely to be attended at birth than women living in urban areas (adjusted OR = 0.25 [0.17 – 0.37]).

Women from households with an average and poor wealth index were less likely to have a birth attended by skilled health personnel than women from wealthy households (adjusted OR = 0.43; CI = [0.31 – 0.59]); (adjusted OR = 0.29; CI = [0.22 – 0.41]).

In terms of profession, women with business or other partners were less likely to benefit from assisted childbirth than their counterparts (adjusted OR = 0.56; CI = [0.42 – 0.74]).

However, women who had performed four or more ANCs (OR = 12.10; CI = [8.24 – 17.77]) and those who had performed 1–3 ANCs (adjusted OR = 6.60; CI = [4.58 – 9.5]) were 12 times and six times more likely to be attended at birth by skilled health professionals than their counterparts who had not performed any ANCs.(Table 3).

## Discussion

The results of this study show that the proportion of skilled births among women who had at least one live birth between 2016 and 2018 was 57.3%. Taken by year, this proportion was 61% in 2016, fell to 59% in 2017 and 50% in 2018. Among women giving birth at home, 19% were also attended by skilled health personnel. The main factors associated with skilled birth attendance were women’s level of education, residence, men’s occupation, number of ANCs performed, wealth index and region of residence. These results have important policy and practical implications.

The proportion of skilled birth attendants in the post-Ebola period (2016–2018) was higher than that reported by the DHS (55%) combining the pre-, intra- and post-Ebola periods (2013–2018).

However, this proportion had an annual downward trend in the post-Ebola period. A study conducted in Sierra Leone in 2019 reported a higher proportion of assisted deliveries in the post-Ebola period, compared with the period before and during Ebola.^23^ The increase in the proportion of skilled birth attendants in the post-Ebola period could be linked to the interventions put in place to strengthen the post-Ebola health system.^23^ Indeed, during the Ebola epidemic, the use of maternal health services declined in the country.^24^ In the face of this ordeal, the government and its partners deployed community awareness-raising interventions and the availability of family planning products to improve the supply and use of these services, including maternal health services. Moreover, in the post-Ebola period, the proportion of births has been on a downward trend, which could be explained by the gradual slackening of efforts to improve the use of services, such as awareness campaigns at the community level and poor communication about the availability of these health services. It would be crucial to maintain and strengthen the interventions deployed in the aftermath of the pandemic, to ensure the availability of health workers in health facilities and to strengthen training supervision of health providers and finally to ensure community health workers’ remuneration.

Women with no formal education were less likely to have skilled births than women with secondary or higher education. Our results were similar to those reported in New Guinea in 2021.^3^

Our results were similar to the study reported by Seidu et al. in New Guinea in 2021^3^ by Fekadu et al. in Ethiopia in 2019^25^ and the one carried out in three West African countries by Edward et al. in 2020,^26^ who reported, respectively, that the chance of having a birth attended by skilled health personnel was higher among women who had a secondary or higher level of education compared to women who had no level of education. This is linked to the fact that, in Guinea, knowledge of sexual and reproductive health is taught from secondary school onwards. The higher a woman’s level of education, the greater her chances of accessing information, and the better able she is to decide for herself who will manage her delivery. Joint efforts to strengthen women’s education policies have helped to increase the school enrolment rate among women, thereby facilitating their access to maternal health services, especially skilled birth services which would help to reduce the risk of childbirth-related complications.^27^ It would be necessary to support the strengthening of school enrolment policies for young girls and to promote literacy policies for mothers in each village.

The study also found that rural women were less likely to be attended by skilled health professionals than urban women. This finding is consistent with studies conducted in 2020 in Ghana^28^ and Ethiopia.^29^ One of the possible reasons for not using skilled birth services is the remoteness of many villages in Guinea and their poor coverage of health services by health workers.^27^ It would be important to deploy more healthcare providers in rural areas and to train community health workers to offer skilled birth attendance services in these areas. It would also be important to promote the extension of health facilities for maternal and neonatal care in rural areas and to provide these primary health care facilities with functional ambulances.

Women who had performed four or more ANCs were more likely to be attended at birth by skilled health professionals than those who had not performed any ANC. Our results are consistent with those of previous studies conducted in Tanzania^30^ and Ethiopia in 2019.^31^ A possible explanation for this association is the good collaboration between women and health care providers during ANC visits, during which women receive more information and knowledge about the advantages and disadvantages of assisted childbirth. Non-attendance at ANC services could result in the failure to detect possible complications before delivery, contributing to an increase in the maternal and neonatal mortality rate. Therefore, it would be necessary to ensure access to maternal health care services in the community, strengthen policies on advanced ANC strategies and ensure the effective application of free delivery policies.

### Limitations and strengths

This study has certain limitations: A possible information bias cannot be excluded as women’s designation of the type of health personnel who assisted with delivery was not verified, the cross-sectional nature of the study, which limits causal inferences, potential residual confounding because of unmeasured factors and finally the focus on a specific time period (2016–2018), which may not reflect long-term trends. Nevertheless, one of the strengths of the study is that it includes data that are representative of the national level of the Guinean population. The study also addresses an identified national operational research priority in that these results could be used to inform policy makers about future trends in assisted childbirth and serve as a basis for advocacy.

### Implication for research and practice

This research has been instructive in highlighting some of the important factors related to skilled birth attendance. These results will serve as a guide for decision-makers in implementing promising strategies for strengthening the healthcare system. One such strategy would be to deploy more health workers in rural areas, training community health workers to provide skilled birth attendance services. It would also be important to provide primary healthcare facilities with functional ambulances. Other recommendations are to make the free care policy for maternal health services effective and to strengthen outreach ANC strategies.

Future cohort research to better understand the benefits of using maternal health services in general and in particular skilled attendance at birth is an important step in reducing maternal mortality.

## Conclusion

The proportion of assisted births reported in this study was 57.3% over the period 2016–2018. This proportion showed an annual downward trend over these 3 years. Women’s level of education, residence and distance from the health facility were associated with skilled birth attendance.

To better understand the complex factors influencing skilled birth attendance in the post-Ebola period and to develop targeted interventions, further research is needed.

It would therefore be important to maintain and improve interventions aimed at strengthening the maternal health system in the post-epidemic period, including the deployment of health providers in rural areas and capacity-building for community health workers.
